# Synthetic hydroxyapatite: a recruiting platform for biologically active molecules

**DOI:** 10.1080/17453674.2019.1686865

**Published:** 2019-11-04

**Authors:** Deepak Bushan Raina, Yang Liu, Hanna Isaksson, Magnus Tägil, Lars Lidgren

**Affiliations:** a Faculty of Medicine, Department of Clinical Sciences, Orthopedics, Lund University, Lund;; b Department of Biomedical Engineering, Lund University, Lund, Sweden

## Abstract

Background and purpose — Targeted delivery of drugs is important to achieve efficient local concentrations and reduce systemic side effects. We hypothesized that locally implanted synthetic hydroxyapatite (HA) particles can act as a recruiting moiety for systemically administered drugs, leading to targeted drug accretion.

Methods — Synthetic HA particles were implanted ectopically in a muscle pouch in rats, and the binding of systemically circulating drugs such as zoledronic acid (ZA), tetracycline and ^18^F-fluoride (^18^F) was studied. The local biological effect was verified in an implant integration model in rats, wherein a hollow implant was filled with synthetic HA particles and the animals were given systemic ZA, 2-weeks post-implantation. The effect of HA particle size on drug binding and the possibility of reloading HA particles were also evaluated in the muscle pouch.

Results — The systemically administered biomolecules (ZA, tetracycline and ^18^F) all sought the HA moiety placed in the muscle pouch. Statistically significant higher peri-implant bone volume and peak force were observed in the implant containing HA particles compared with the empty implant. After a single injection of ZA at 2 weeks, micro HA particles showed a tendency to accumulate more ^14^C-zoledronic acid (^14^C-ZA) than nano-HA particles in the muscle pouch. HA particles could be reloaded when ZA was given again at 4 weeks, showing increased ^14^C-ZA accretion by 73% in microparticles and 77% in nanoparticles.

Interpretation — We describe a novel method of systemic drug loading resulting in targeted accretion in locally implanted particulate HA, thereby biologically activating the material.

In drug delivery, one important goal is to achieve efficient tissue concentrations in targets known for poor drug penetration. Local drug delivery can be one solution and may involve a carrier, able to act as a temporary depot to release the active biomolecules (Raina et al. 2016). The possibility of reloading such a carrier has until now not been described. Furthermore, a local delivery approach most often requires surgery. Targeted delivery of drugs by coupling them to tissue specific ligands, the so-called ligand–receptor interaction, is an example of a systemic approach to enhance drug concentration, yet the efficiency is less than 10% and it still involves complicated fabrication processes (Kirpotin et al. 2006, Bae and Park 2011).

We propose implanting a recruiting and reloadable particulate apatite moiety, within the tissue of interest, to which systemically administered drugs circulating in the bloodstream could bind due to a high chemical affinity. A biomaterial in the form of particulate HA embedded in calcium sulphate (CaS) allows for in-situ setting. Based on the affinity to HA, there are antibiotics today in clinical use for bone infection that could be candidates for seeking HA as a recruiting moiety (Perrin 1965). By activating the ceramic material, it can initially exert a local antibacterial effect and later be reloaded via systemic administration. We hypothesized that particles of synthetic HA possess binding sites such as calcium, phosphate and hydroxyl groups, which when placed in a targeted tissue can act as recruiting moiety for systemically administered biomolecules.

The primary aim of our study was to demonstrate whether a systemically administered bisphosphonate, zoledronic acid (ZA) with known affinity to HA, could be bound to synthetic particulate HA implanted in an ectopic location. Second, our aim was to prove a biological effect of the drug-seeking phenomenon in bone, by using a fenestrated implant containing HA particles in an orthotopic model in rats. Additionally, we present: (1) an evaluation of the effect of the HA particle size on the drug binding capacity in an ectopic implantation model; (2) an assessment of the possibility of reloading the implanted HA particles via systemic delivery of a model drug and (3) an exploration of other binding agents, given systemically, such as an antibiotic, tetracycline, and a radioactive tracer ^18^F, and their ability to seek local HA.

## Methods

### Study design

**Figure 1.**
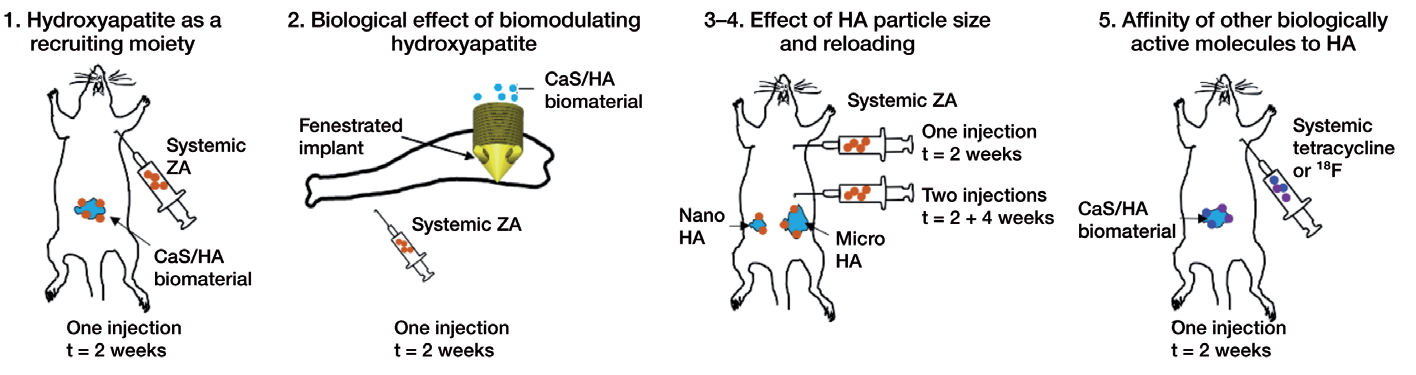
Overview of the experimental design used in the study.

The observations made in this study are based on in-vivo experiments carried out on the laboratory rat as a model biological system. The first study describes the uptake of a bisphosphonate, zoledronic acid (ZA), in a biphasic calcium sulphate (CaS)/hydroxyapatite (HA) based biomaterial. The material was implanted in an abdominal muscle pouch model, an ectopic non-osseous site, without the presence of living bone (Raina et al. 2016). To verify the biological effects, an implant integration model was used in rats. The next experiment was performed to evaluate the effect of the hydroxyapatite particle size on uptake of ZA in the ectopic muscle pouch model. The final experiments were performed to evaluate the affinity of 2 other drug classes to synthetic HA including the antibiotic tetracycline and positron-emitting radioactive tracer ^18^F (Figure 1).

### Uptake of ZA in ectopically implanted CaS/HA biomaterial pellets: hydroxyapatite as a recruiting moiety

CaS/HA biomaterial pellets were prepared by mixing 1 g of CaS/HA pre-mixed powder (60% CaS and 40% HA) with 0.43 mL non-ionic radiographic contrast agent (Iohexol) using a sterile spatula. The slurry was transferred to a 1 mL graduated syringe and 40 µL of the paste was poured into each well of a sterile nylon mold (Ø = 5 mm). 12 pellets were casted and each pellet contained approximately 80 mg CaS/HA. The contents of each well were allowed to set for 30 min and the pellets were retrieved from the mold. The entire process was performed in a sterile environment under a laminar airflow bench. 6 male Sprague-Dawley (SD) rats (average weight 358 g) were used for the experiment. 4 rats received 1 pellet of CaS/HA biomaterial each in the abdominal muscle following an established protocol described in detail by our group previously (Raina et al. 2016). The remaining 2 animals were operated the same way without receiving the CaS/HA biomaterial pellet (SHAM). After 2 weeks, all animals were given a subcutaneous injection of ^14^C labelled ZA (concentration: 1 mg/mL, specific radioactivity: 7.1 MBq/mL, radiochemical purity: > 95%) at a dose of 0.1 mg/kg, a standard dose in rodent experiments (Amanat et al. 2007). After a period of 24 h, all 6 animals were killed by CO_2_ asphyxiation. 4 pellets of CaS/HA biomaterial were retrieved from the biomaterial implanted animals. A 4-mm biopsy of the muscle (n = 2) was harvested from the SHAM operated animals from the same anatomical location. Samples were individually placed in 5 mL scintillation tubes and immersed in 2 mL of 5M HCl for 48 h at room temperature to aid in rapid decalcification and softening. All samples were homogenized to form a slurry using an ultrasound-based tissue homogenizer (1 min/sample). 0.5 mL of the slurry was mixed with 4.5 mL scintillation cocktail (Optiphase, HiSafe 2, PerkinElmer, Waltham, MA, USA), homogenously mixed and read using a scintillation counter (Wallac 1414, PerkinElmer, USA).

### Implant integration model: biological effect of biomodulating hydroxyapatite

Medical grade polyether ether ketone (PEEK) implant (outer Ø = 3.5 mm, inner Ø = 2.1 mm, hole Ø = 1.4 mm and height = 6.3 mm) was custom made in the form of a threaded hollow cylindrical core with a conical frustum at the bottom and contained 3 equally spaced holes. A PEEK implant was used over conventional titanium to avoid metal artefacts seen using X-ray-based imaging of metallic implants. A detailed description of the chamber model is mentioned elsewhere (Raina et al. 2019). In brief, a 3.2 mm Ø hole was created in the right proximal tibia of rats just under the tibial epiphysis. The implant was placed press-fit in the metaphyseal bone and screwed with the aid of a custom-made screwdriver. 2 groups were used for evaluation of bone–implant anchorage; (1) Empty PEEK implant (control) and (2) PEEK implant filled with a CaS/HA biomaterial. The control group involving the use of the empty implant has also been described earlier in our recent study and data are used for comparison only (Raina et al. 2019). 22 male SD rats were divided into 2 equally sized groups (average weight = 378 g, n = 11/group). After 2 weeks, the animals receiving implants containing CaS/HA biomaterial were injected with a single subcutaneous dose of ZA (concentration: 0.8 mg/mL, injected dose: 0.1 mg/mL). 6 weeks post-surgery, the animals were killed using CO_2_ asphyxiation and the harvested tibiae were evaluated for peri-implant bone formation using radiography, microcomputed tomography (micro-CT), mechanical testing, and histology.

Radiography and Micro-CT imaging was performed using a NanoScan micro CT scanner (Mediso Medical Imagining System, Budapest, Hungary) to obtain images with an effective voxel size of 10 µm (X-ray voltage: 65 kV, current: 123 µA, exposure: 1300 ms). Peri-implant bone formation was measured immediately around the implant holes within the medullary canal and expressed as bone volume (BV) based on established protocols (Raina et al. 2019).

Mechanical testing was performed on an Instron® (8511) biaxial load frame (Instron, Norwood, MA, USA) connected to a 250N load sensor. Samples were mounted on a custom-made jig. All specimens were subjected to a pre-loading protocol for 10 s before a total pull-out was performed. A load rate of 0.5 mm/s was used for the pull-out and the force-displacement curves were used to obtain the peak force (Raina et al. 2019).

Routine procedures for decalcified histology were followed. Briefly, tissue fixation was done in 4% neutral buffered formalin solution overnight following which EDTA (10% w/v) based decalcification was carried out for 5 weeks before paraffin embedding. Sections of 5 µm thickness were cut and stained with H&E by following the manufacturer’s guidelines (Thermo Fisher Scientific, Waltham, MA, USA).

### Role of hydroxyapatite particle size on drug accumulation and the possibility of reloading

Commercially available HA particles of micrometer size (10 µm, Sigma-Aldrich, Product Number: 900203; Sigma-Aldrich, St Louis, MO, USA) and nanometer size (< 200 nm, Sigma-Aldrich, Product Number: 677418) were sterilized by autoclaving. For the uptake experiment, 25 mg of both particle sizes were weighed. 18 male SD rats (average weight: 326 g) were operated in the abdominal muscle pouch and each rat received loose nano-HA (nHA) particles on the right side of the abdominal midline and loose micro-HA (mHA) particles on the left side of the abdominal midline. Particles were introduced into the muscle pouch by using a sterile pipette tip as a funnel. The muscle was sutured using 2 single non-resorbable sutures, which also aided in locating the particles at harvest. At 13 days post-surgery, all animals received a single subcutaneous injection of ^14^C labelled ZA (concentration: 1 mg/mL, specific radioactivity: 7.1 MBq/mL, radiochemical purity: > 95%) at a dose of 0.1 mg/kg (animal weight averaged to 400 g). 1 day after the injection, 6 animals were killed and the radioactive counts were measured by following the protocol described in section 2.2. At 27 days post-surgery, 6/12 animals received a repeated subcutaneous injection of ^14^C labelled ZA (dose: 0.1 mg/kg, animal weight averaged to 400 g). 1 day after the second injection, all remaining 12 animals were killed to evaluate the efficacy of reloading the HA particles with ZA. The 6 animals that were injected on day 13 and killed at t = day 28 were used as controls for the reloading experiment.

### Evaluating the affinity of other biologically active molecules to hydroxyapatite

The antibiotic tetracycline and ^18^F-fluoride radioactive isotope were the other 2 biologically active molecules whose ability to systemically seek and bind hydroxyapatite was evaluated. Pellets of CaS/HA biomaterial were prepared by following the same procedure as described earlier. 4 male SD rats (average weight 361 g) were used and each animal received 1 single pellet of the CaS/HA biomaterial in the abdominal muscle. After 2 weeks, 2 animals received a single subcutaneous injection of tetracycline (dose 20 mg/kg) and the animals were killed 1 day later. The pellets were harvested from the muscle pouch, carefully cleaned of all surrounding muscle/connective tissue and formalin fixed overnight followed by routine histological preparation. Tissue sections were placed on glass slides for fluorescence microscopy. The remaining 2 animals were injected with Na-^18^F via the tail vein 2 weeks after CaS/HA biomaterial implantation (specific radioactivity: animal 1: 85 MBq and animal 2: 120 MBq) while maintaining isoflurane anesthesia (2% isoflurane and 1:1 mixture of O_2_ and N_2_O, flow rate: 0.4 L/min). After a waiting period of 1 h, the animals were placed in a positron emission tomography scanner (NanoPET/CT, Mediso Medical Imagining Systems, Hungary) and subjected to micro-CT to detect the anatomical location of the implanted pellet (projections: 240, scan: semi-circular, X-ray voltage: 65 kV, exposure: 500 ms, voxel size: 141 µm). Imaging setup described earlier by Mathavan et al. (2019) was used. The uptake of Na-^18^F in the CaS/HA biomaterial was analyzed using PET imaging and a voxel size of 0.4 mm was achieved. The CT and PET projections were overlapped to confirm the tracer uptake in the CaS/HA biomaterial.

### Statistics

Mann–Whitney U-test was used to compare two groups. Paired data analysis was performed using Wilcoxon’s signed rank test. Data in the graphs are shown as mean ± SD.

### Ethics, funding, and potential conflicts of interest

All animal experiments were approved by the Swedish board of agriculture (Permits: M124-14 and M79-15). The ARRIVE guidelines have been followed to provide details regarding in-vivo experiments involving the usage of laboratory animals. VINNOVA, the Swedish agency for innovation systems (Grant number: 2017-00269), the Swedish Research Council (Vetenskapsrådet, Grant number: 2015-06717) and the Alfred Andersson foundation funded this study. LL is a board member of Bone Support AB, Sweden and Ortho Cell, Australia. MT and DBR have received options from Ortho Cell, Australia for work unrelated to this study.

## Results

### Uptake of 14C-ZA in the CaS/HA biomaterial at an ectopic location

**Figure 2.**
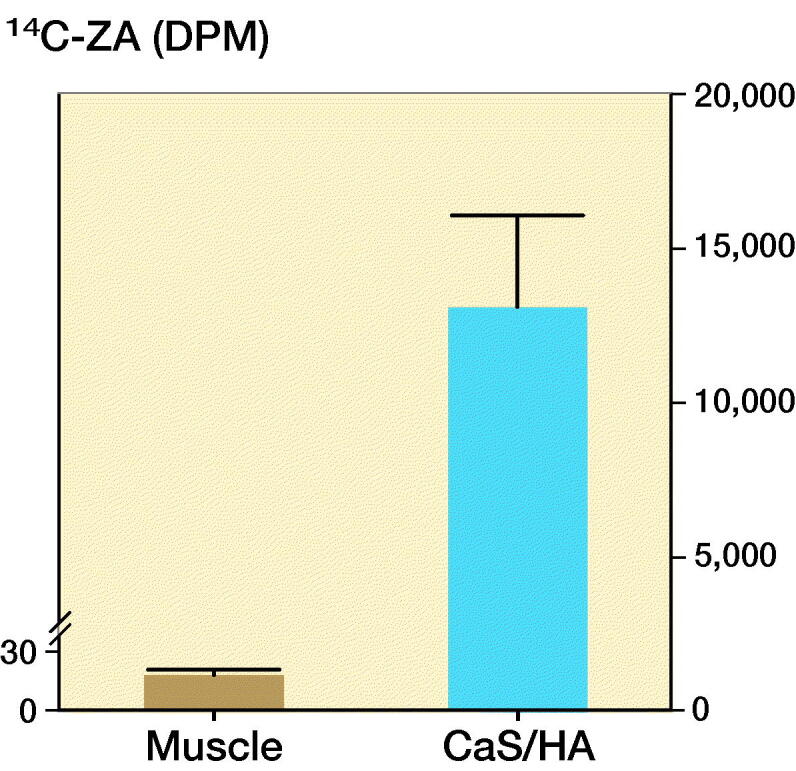
Uptake of ^14^C-ZA in the CaS/HA biomaterial placed in the abdominal muscle, 1 day post subcutaneous administration of ^14^C-ZA. Observe the difference in the scale of the y-axes for the 2 groups. Bars indicate uptake of ^14^C-ZA in the muscle (brown) and CaS/HA biomaterial (blue).

^14^C-ZA uptake was confirmed in the pellet of CaS/HA biomaterial placed in the abdominal muscle pouch using scintillation counting (Figure 2). Negligible counts were detected in the abdominal muscle (muscle: 18 DPM vs. only cocktail: 8 DPM) of the control animals that were injected with only ^14^C-ZA.

### Implant integration model: biological effect of biomodulating hydroxyapatite

Representative radiographs, micro-CT slices and the quantitative analysis of BV measured using micro-CT indicated significantly higher BV in the Implant + CaS/HA + systemic ZA group compared with the empty implant (p < 0.001) (Figure 3). Representative histology images corroborated the micro-CT results well and a higher amount of viable bone tissue around the implant was found in the systemic ZA group, compared with the empty control both at low and high magnifications. Furthermore, the higher BV fraction around the implant in the systemic ZA group also resulted in better implant osseointegration (increased pull-out peak force) as seen from the increased peak force in the pull-out testing (p = 0.008).

Figure 3.Biological effect of hydroxyapatite biomodulation assessed in the implant integration model in rats. Top panel shows representative radiograph, micro-CT slice, histology overview (H&E staining), and high magnification histology (H&E staining), respectively from the Implant + empty group while the middle panel shows images from the Implant + CaS/HA + systemic ZA group. Bottom left indicates micro-CT quantification of bone volume (BV) immediately around the implant holes and bottom right shows peak pull-out force. In the histology images * indicates new bone formation around the implant. Scale bar in the overview histology images represents 1 mm and magnified images represents 100 µm. **^a^**indicates p < 0.001 and **^b^**indicates p < 0.01 (Mann–Whitney U-test). Data for the empty group are taken from an earlier study for comparison (Raina et al. 2019).

**Figure:**
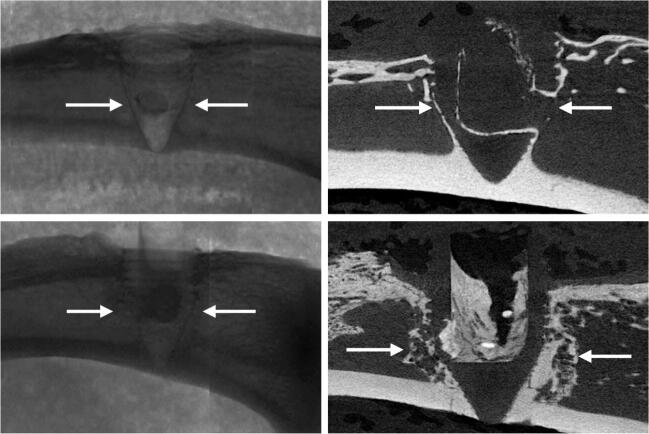

**Figure:**
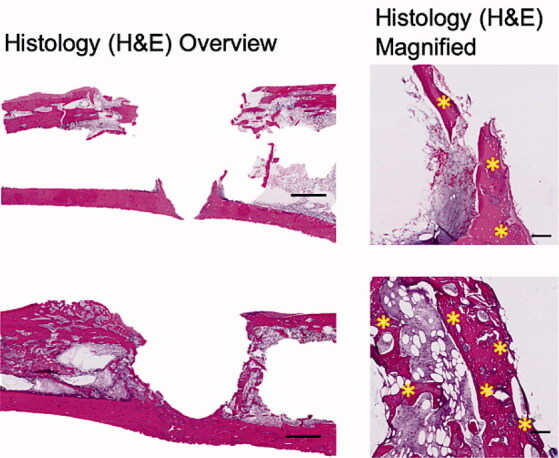

**Figure:**
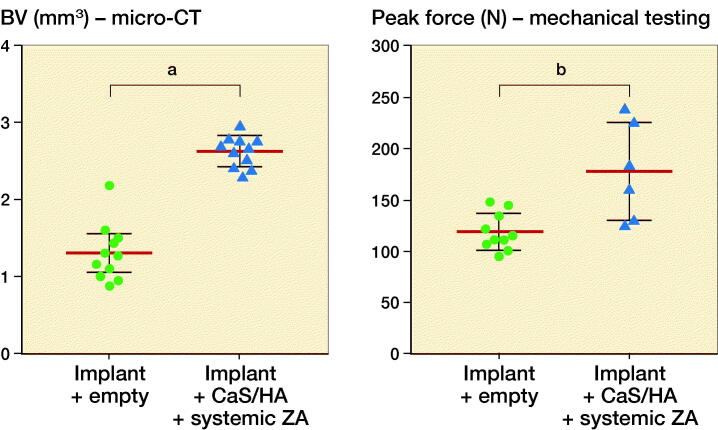

### Effect of hydroxyapatite particle size on 14C-ZA uptake

**Figure 4.**
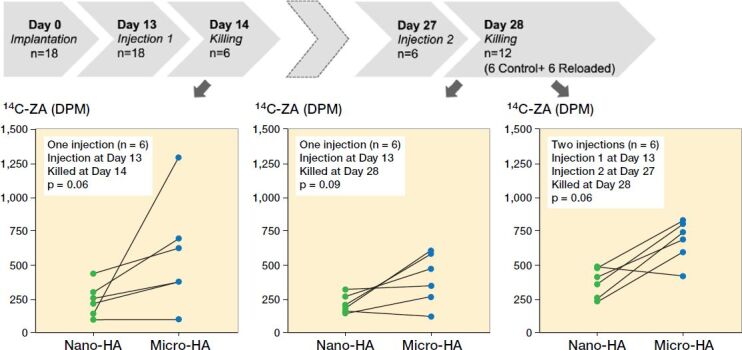
Timeline and uptake kinetics of ^14^C-ZA in micro- and nano-sized HA particles placed in the abdominal muscle pouch. Left: Uptake of ^14^C-ZA in the animals injected with ^14^C-ZA on day 13 and killed on day 14. Middle: Uptake of ^14^C-ZA in the animals injected with ^14^C-ZA on day 13 and killed on day 28. Right: Uptake of ^14^C-ZA in the animals injected with ^14^C-ZA on day 13 and day 27 and killed on day 28. Each line on the graph presents paired data from the same animal. Statistical analysis was performed using Wilcoxon’s matched pairs signed rank test.

A higher ^14^C-ZA uptake was observed in micro-sized HA particles as compared with the nano-HA particles (p = 0.06) (Figure 4, left).

### Possibility of reloading hydroxyapatite particles

**Figure 5.**
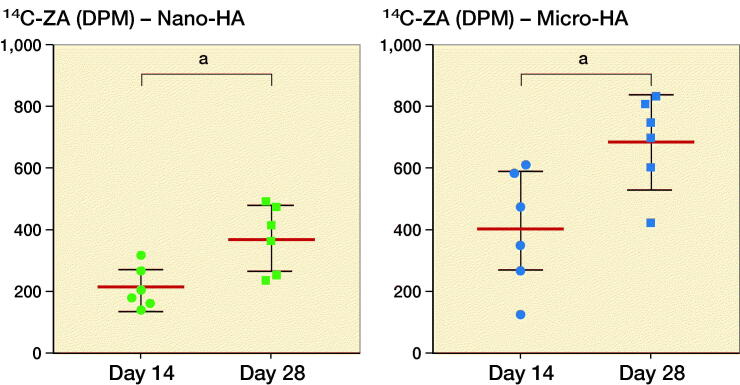
Reloading efficiency of HA particles with ^14^C-ZA. Nano- (left) and micro-sized (right) HA particles injected with ^14^C-ZA and its uptake after 1 or 2 subcutaneous injections of ^14^C-ZA. **^a^** indicates p < 0.05 (Mann–Whitney U-test).

There was a marked difference between the uptake of ^14^C-ZA after 1 and 2 subcutaneous injections of ^14^C-ZA, indicating the possibility of reloading HA particles on a need-be basis (Figures 4 and 5). Similar to the animals killed at day 14 (Figure 4, left), the animals that received 1 subcutaneous dose of ^14^C-ZA on day 13 followed by killing on day 28 (Figure 4, middle), showed an increased ^14^C-ZA uptake in microparticles compared with nanoparticles (p = 0.09) with a further increase after 2 subcutaneous injections (Figure 4, right). By administering ^14^C-ZA on 2 occasions instead of 1, a 77% increase in the average uptake of ^14^C-ZA was noted in the nanoparticles (p = 0.02) while micro-HA particles exhibited an increase of 73% (p = 0.02) (Figure 5).

### Uptake of antibiotic tetracycline and radioemitter 18F in CaS/HA biomaterial

**Figure 6.**
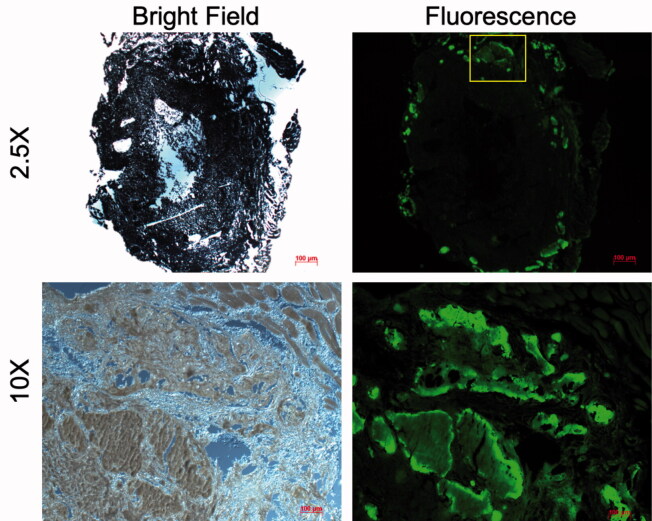
Uptake of tetracycline (administered systemically) in a pellet of CaS/HA biomaterial placed in the abdominal muscle pouch of rats, 24 h post-administration detected using fluorescence microscopy. Yellow box (top, right) indicates the approximate region where the high magnification (bottom, right) fluorescence image was captured. Low magnification image was captured with an exposure of 1 s while the high magnification image was captured with an exposure of 200 ms.

Fluorescence microscopy images confirmed the uptake of the antibiotic tetracycline in the CaS/HA biomaterial after subcutaneous administration of tetracycline 2 weeks post-pellet implantation and subsequent imaging 24 h later (Figure 6). Importantly, most of the fluorescent signal was seen on the periphery of the CaS/HA biomaterial with very limited fluorescence detected in the middle of the specimen.

**Figure 7.**
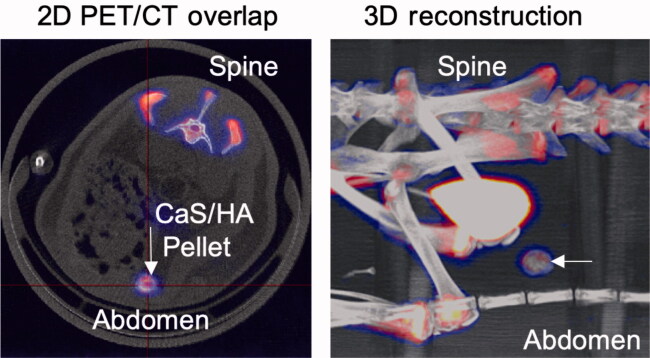
Uptake of ^18^F (administered systemically) in a pellet of CaS/HA biomaterial placed in the abdominal muscle pouch of rats, 1 h post-administration detected using PET-CT.

Using PET-CT, the uptake of the radioemitter ^18^F in the CaS/HA biomaterial ectopically placed in the abdominal muscle pouch was shown. Apart from the CaS/HA biomaterial, the uptake was also observed in other hard tissues. A substantial amount of the tracer was cleared by the kidneys and found in the urinary bladder (Figure 7).

## Discussion

We have shown that synthetic HA particles can be used as a recruiting moiety for different drug classes administered systemically and their affinity to HA binding sites can activate the particulate material to exert a biological effect. After systemic administration of radioactive ZA, we found an accretion in the synthetic HA particles implanted in the abdominal muscle pouch, which remained for at least 2 weeks after injection. Similar results have been observed in an orthotopic model in osteoporotic rats for up to 6 months (unpublished results). The ZA not only seeks but also modulated and activated HA, thereby enhancing bone anchorage as shown in the implant integration model. The timing of the injection may be critical, and has been established to be between 1 and 2 weeks in rat models of fracture healing (Amanat et al. 2007). This time interval theoretically fits well within the time frame reported for HA deposition in a fracture callus collagen network, and with the fluid mechanics necessary for drug transport (Andreshak et al. 1997, Amanat et al. 2007, Mathavan et al. 2019).

The use of the biomodulation framework is promising also for other drug classes, apart from obvious bone-seeking drugs like bisphosphonates. Using particulate HA as a recruitment platform may also open up new treatment methods in infections and tumors. Tetracycline is commonly used for dynamic histomorphometry of bone and Perrin in 1965 confirmed that tetracycline binds to HA (Perrin 1965). Furthermore, Bernhardsson et al. (2018) recently showed uptake of a positron-emitting tracer ^18^F in an ectopic location in rats, which contained pellets of allogenic bone or granular HA. While the literature reveals that certain drug classes have affinity for bone or HA in general, none of the studies have looked at the relationship of physical properties of HA, such as particle size and its effect on drug-binding capacity, especially in in-vivo models. More so, our study also presents a novel concept of reloading the implanted HA particles, which opens possibilities for on-demand additional local drug targeting. Owing to a larger surface area, we hypothesized that nanoparticles in comparison with microparticles could theoretically give access to an increased number of binding sites and thus higher drug accretion. However, our findings indicate the contrary, which could be because the intra-particulate open space is larger between microparticles compared with the densely packed nanoparticles, making the binding sites more easily accessible. Another explanation could be that the biological clearance of the nanoparticles from the tissue locally is more rapid, with fewer total available particles and thus reduced binding affinity. This observation agrees with an earlier study, which compared the effect of polymer based micro- and nanoparticle delivery in the rodent peritoneum (Kohane et al. 2006). The authors could detect microparticles in the peritoneum for up to 14 days while the nanoparticles were cleared as early as 2 days with substantial uptake in the spleen and the liver.

Local HA, in the form of micro- to nanoparticles at the site of infection, could act as a carrier but also as a recruiting moiety for systemically administered HA binding antibiotics. The affinity of each antibiotic would foremost depend on its chemical structure, i.e., binding capacity to calcium, phosphate, and hydroxyl groups and HA amount and size. It is compelling that some of the recommended second-level systemic antibiotics for PJI such as rifampicin, tetracycline, and daptomycin all have a chemical structure that would allow them to bind to apatite. A novel approach for a biphasic ceramic could be to have the antibiotic not only embedded in the soluble sulphate phase but also, by using millions of nano-HA particles already functionalized with antibiotics, to provide extended sustained local antibiotic delivery. The HA particles could then further be reloaded by systemic administration with the same or a different antibiotic that has high affinity for HA. Nanoparticles of HA are shown to be internalized by several cell types (Yuan et al. 2010, Zhao et al. 2018), which could give the possibility of intracellular drug delivery to eradicate bacteria known to reside within the cells.

Apart from antibiotics, Lewington (2005) in a review on bone-seeking radioactive isotopes describes a group of isotopes such as ^32^P, ^89^Sr, ^186^Re, and ^223^Ra possessing high affinity to metabolically active bone. De Klerk et al. (1992) combined ^186^Re with hydroxyethylidene diphosphonate to form a bisphosphonate complex with an aim to achieve high bone accretion for treatment of metastatic bone disease. Even bisphosphonates have been described as carriers for various tumor-targeting radioisotopes for diagnostic purposes as well as for pain management in tumors (Palma et al. 2011). In this study, we used a model radioemitter ^18^F to verify accretion in synthetic HA particles. Based on the literature, other clinically relevant isotopes could also be systemically administered and concentrated in a target tissue implanted with large number of nano- and micro-HA particles for theranostic treatment of locally aggressive tumors or solitary metastasis. Agents that exhibit strong affinity to hydroxyapatite can also be coupled with other therapeutic agents (Ramanlal Chaudhari et al. 2012) wherein the molecule with affinity acts as a guide and takes the drug to the target tissue.

## Conclusion

This study recapitulates some of the early studies with systemically administered agents traced in bone and hydroxyapatite (HA). A systemically administered bisphosphonate, ZA, seeks HA acting as a recruiting moiety. A fenestrated bone-anchored PEEK implant was filled with synthetic HA microparticles and after a period of 2 weeks a bisphosphonate, zoledronic acid, was systemically administered. The synthetic HA particles acted as a ZA-recruiting biomodulated moiety and resulted in improved bone–implant anchorage, indicating a biological effect. The specific binding of locally implanted HA particles in the muscle was also verified for tetracycline and ^18^F. Our study also shows that the size of the HA particles plays an important role in the binding of a systemically administered drug. Micro-sized particles tend to bind more drug compared with nanoparticles. Systemic loading of synthetic HA particles can be carried out at several predetermined time intervals or on a need-be basis as long as a sufficient amount of HA particles with free binding sites are available at the target tissue.

## Clinical significance

We present a novel concept of targeted drug delivery by providing a HA-based recruiting moiety that can attract certain classes of systemically circulating drugs and lead to their accumulation within the target tissue. A library of biomolecules that have the ability to chemically interact with HA could in future be used for targeted delivery of drugs in scenarios involving bone regeneration, infections, or tumors, and likely be implemented in other tissues.

## References

[CIT0001] Amanat N, McDonald M, Godfrey C, Bilston L, Little D. Optimal timing of a single dose of zoledronic acid to increase strength in rat fracture repair. J Bone Miner Res 2007; 22(6): 867–76. 17371160 10.1359/jbmr.070318

[CIT0002] Andreshak J L, Rabin S I, Patwardhan A G, Wezeman F H. Tibial segmental defect repair: chondrogenesis and biomechanical strength modulated by basic fibroblast growth factor. Anat Rec 1997; 248(2): 198–204. 9185985 10.1002/(SICI)1097-0185(199706)248:2<198::AID-AR6>3.0.CO;2-P

[CIT0003] Bae Y H, Park K. Targeted drug delivery to tumors: myths, reality and possibility. J Control Rel 2011; 153(3): 198–205. 10.1016/j.jconrel.2011.06.001 PMC3272876 21663778

[CIT0004] Bernhardsson M, Sandberg O, Ressner M, Koziorowski J, Malmquist J, Aspenberg P. Shining dead bone: cause for cautious interpretation of [(18)F]NaF PET scans. Acta Orthop 2018; 89(1): 124–7. 28914114 10.1080/17453674.2017.1372097 PMC5810820

[CIT0005] de Klerk J M, van Dijk A, van het Schip A D, Zonnenberg B A, van Rijk P P. Pharmacokinetics of rhenium-186 after administration of rhenium-186-HEDP to patients with bone metastases. J Nucl Med 1992; 33(5): 646–51. 1373767

[CIT0006] Kirpotin D B, Drummond D C, Shao Y, Shalaby M R, Hong K, Nielsen U B, Marks J D, Benz C C, Park J W. Antibody targeting of long-circulating lipidic nanoparticles does not increase tumor localization but does increase internalization in animal models. Cancer Res 2006; 66(13): 6732–40. 16818648 10.1158/0008-5472.CAN-05-4199

[CIT0007] Kohane D S, Tse J Y, Yeo Y, Padera R, Shubina M, Langer R. Biodegradable polymeric microspheres and nanospheres for drug delivery in the peritoneum. J Biomed Mater Res Part A 2006; 77(2): 351–61. 10.1002/jbm.a.30654 16425240

[CIT0008] Lewington V J. Bone-seeking radionuclides for therapy. J Nucl Med 2005; 46 (Suppl. 1): 8s–47s. 15653650

[CIT0009] Mathavan N, Koopman J, Raina D B, Turkiewicz A, Tägil M, Isaksson H. ^18^F-fluoride as a prognostic indicator of bone regeneration. Acta Biomater 2019; 90: 403–11. 30965143 10.1016/j.actbio.2019.04.008

[CIT0010] Palma E, Correia J D G, Campello M P C, Santos I. Bisphosphonates as radionuclide carriers for imaging or systemic therapy. Mol BioSystems 2011; 7(11): 2950–66. 10.1039/c1mb05242j 21879109

[CIT0011] Perrin D D. Binding of tetracyclines to bone. Nature 1965; 208(5012): 787–8. 5868891 10.1038/208787a0

[CIT0012] Raina D B, Isaksson H, Hettwer W, Kumar A, Lidgren L, Tägil M. A biphasic calcium sulphate/hydroxyapatite carrier containing bone morphogenic protein-2 and zoledronic acid generates bone. Sci Rep 2016; 6: 26033. 27189411 10.1038/srep26033 PMC4870695

[CIT0013] Raina D B, Larsson D, Sezgin E A, Isaksson H, Tägil M, Lidgren L. Biomodulation of an implant for enhanced bone–implant anchorage. Acta Biomater 2019; 96:619–30. 31301423 10.1016/j.actbio.2019.07.009

[CIT0014] Ramanlal Chaudhari K, Kumar A, Megraj Khandelwal V K, Ukawala M, Manjappa A S, Mishra A K, Monkkonen J, Ramachandra Murthy R S. Bone metastasis targeting: a novel approach to reach bone using zoledronate anchored PLGA nanoparticle as carrier system loaded with docetaxel. J Control Rel 2012; 158(3): 470–8. 10.1016/j.jconrel.2011.11.020 22146683

[CIT0015] Yuan Y, Liu C, Qian J, Wang J, Zhang Y. Size-mediated cytotoxicity and apoptosis of hydroxyapatite nanoparticles in human hepatoma HepG2 cells. Biomaterials 2010; 31(4): 730–40. 19836072 10.1016/j.biomaterials.2009.09.088

[CIT0016] Zhao H, Wu C, Gao D, Chen S, Zhu Y, Sun J, Luo H, Yu K, Fan H, Zhang X. Antitumor effect by hydroxyapatite nanospheres: activation of mitochondria-dependent apoptosis and negative regulation of phosphatidylinositol-3-kinase/protein kinase B pathway. ACS Nano 2018; 12(8): 7838–54. 30059628 10.1021/acsnano.8b01996

